# Clinical and Radiological Characteristics for Recurrence of Chronic Subdural Hematoma: A Systematic Review and Meta-Analysis

**DOI:** 10.3390/neurolint14030057

**Published:** 2022-08-26

**Authors:** Rakesh Mishra, Harsh Deora, William Andres Florez-Perdomo, Luis Rafael Moscote-Salazar, Ezequiel Garcia-Ballestas, Md Moshiur Rahman, Adesh Shrivastava, Sumit Raj, Vishal Chavda, Nicola Montemurro, Amit Agrawal

**Affiliations:** 1 Department of Neurosurgery, Institute of Medical Sciences, Banaras Hindu University, Varanasi 221005, UP, India; 2 Department of Neurosurgery, National Institute of Mental Health and Neurosciences, Bengaluru 530068, KA, India; 3 Medicina General, Universidad Surcolombiana, ConcejoLatinoamericano de Neurointensivismo, CLaNi, ClinicaSahagún IPS SA, Monteria 230002, Colombia; 4 Department of Neurosurgery, University of Cartagena, Cra. 50 #24120, Cartagena de Indias 130002, Colombia; 5 Centro de InvestigacionesBiomedicas (CIB), Faculty of Medicine, University of Cartagena, Cartagena 130005, Colombia; 6 Department of Neurosurgery, Holy Family Red Crescent, Medical College, Dhaka 1203, Bangladesh; 7 Department of Neurosurgery, All India Institute of Medical Sciences, Saket Nagar, Bhopal 462020, MP, India; 8 Department of Pathology, Stanford School of Medicine, Stanford University Medical Center, Palo Alto, CA 94305, USA; 9 Department of Neurosurgery, Azienda Ospedaliera Universitaria Pisana (AOUP), University of Pisa, 56100 Pisa, Italy

**Keywords:** recurrent chronic subdural hematoma, cSDH, radiological parameters, neurosurgery, elderly, traumatic brain injury

## Abstract

Chronic subdural hematoma (cSDH) is one of the most studied clinical entities in the neurosurgical literature. Management of cSDH is complicated by its propensity to recurrence. Various factors for the development of recurrence of cSDH have been described in various clinical, epidemiological, and observational studies, yet the evidence available is limited. A systematic review and meta-analysis as per PRISMA guidelines to identify clinical and radiological factors which can predict the development of recurrence in cSDH. A total of 14 studies were included for the systematic review and meta-analysis after a comprehensive search of the online databases. Eight studies were of high methodological quality. Age, use of anticoagulants, obesity, seizure, and liver disease were found to be statistically significant clinical risk factors for the development of recurrence in cSDH. Among the radiological parameters, the internal structure of the hematoma and the width of the hematoma was found to be significant risk factor predicting the development of recurrence. Age >75 years, use of anticoagulation therapy, liver disease, and obesity were significant risk factors for cSDH recurrence. Pneumocephalus, internal architecture of hematoma, bilateral cSDH, the width of hematoma, and the presence of bilateral cSDH are important radiological parameters of the development of recurrent cSDH

## 1. Introduction

Chronic subdural hematoma (cSDH) is a common neurosurgical disease, particularly in the elderly. The most challenging management in these patients is the recurrence of the subdural collection after apparently adequate surgery. There have been several studies that analyze the risk factors associated with the recurrence of cSDH. However, the studies present inconsistent and contradictory results; therefore, the evidence on risk factors for cSDH recurrence is not clear. This systematic review aims to determine various clinical and radiological risk factors that are predictors of the recurrence of cSDH.

## 2. Methods

### 2.1. Database Search

We searched for all available studies on recurrent cSDH in various online databases, as described below. A literature search for randomized controlled trials (RCTs), non-RCTs, prospective and retrospective cohort studies was conducted on the following databases to find the relevant articles up to the year 2019: PUBMED (until 2019), Specialized Registry Cochrane Injuries Group (until 2019), Central Cochrane Registry of Controlled Trials (The Cochrane Library), MEDLINE (Ovid), EMBASE (Ovid), PubMed. Additionally, the reference list of included studies was added as potentially eligible studies.This systematic review was developed and reported in accordance with the Preferred Reporting Items for Systematic Review and Meta-analysis (PRISMA) guidelines. The approval code is 355196.

### 2.2. Criteria for Online Search

The studies to be included were screened separately using the following inclusion criteria: (1) patients with cSDH and (2) RCT, non-RCT, prospective, and retrospective cohort studies describing various risk factors.

The following keywords were used for the search: (Chronic subdural hematoma) OR (Subdural Hematomas, Chronic) OR Chronic subdural hemorrhage) AND (Risk factor [Mesh Terms] OR Population at risk) AND (recurrence OR Relapses) “Recurrent subdural hematoma” OR “ chronic subdural hematoma” OR “recurrent subdural hemorrhage” OR “spontaneous subdural hemorrhage” OR “traumatic chronic subdural hemorrhage” OR (“spontaneous intracranial hemorrhage” AND “subdural hematoma” NOT “intracerebral hematoma” NOT “subarachnoid hemorrhage” NOT “aneurysmal ruptured”) OR (“traumatic brain injury” AND “intracranial hemorrhage” AND “subdural hematoma” NOT “intracerebral hematoma” NOT “subarachnoid hemorrhage” NOT “traumatic subarachnoid hemorrhage”) AND (“Risk factor” OR “population at risk”) AND “recurrence risk”.

### 2.3. Inclusion Criteria for Studies

All the studies were subjected to screening for eligibility based on the recommendations of the meta-analysis and the systematic reviews of the PRISMA declaration for the presentation of the systematic reviews, the meta-analyses, and the Cochrane manual of systematic reviews and meta-analyses.

### 2.4. Exclusion Criteria

The search was limited to human studies and English publications. Case reports and studies with fewer than ten (n = 10) patients, genetic studies, and studies with recommendations and guidelines were excluded. Studies for which the complete text could not be retrieved were also excluded.

### 2.5. Evaluation of the Quality of the Studies Included

The study was carried out using the Newcastle–Ottawa Quality Assessment Scale, and studies with scores of 6 or more were considered of high methodological quality. Those with scores in the range of 4 to 5 were considered moderate Quality.

### 2.6. Analysis of Data

#### 2.6.1. Clinical Risk Factors

Data extracted from the studies as clinical risk factors predicting recurrence of cSDH included: age, sex, seizures, smoking, hypertension, diabetes, heart kidney, and liver disease, alcohol abuse, antiplatelet or anticoagulant drugs, stroke, and head trauma.

#### 2.6.2. Radiological Risk Factors

Data extracted from the included studies for radiological risk factors predicting the recurrence of cSDH included: unilateral or bilateral cSDH, the width of the hematoma, midline shift, and internal architecture of the hematoma density ((homogeneous, laminar, separated, trabeculated). Wherever data were missing authors were contacted and some doubts were clarified by consensus.

#### 2.6.3. Statistical Methods Used

Statistical analysis was carried out using the relative risk with the generic inverse methodology of the variance (IV) to combine the odds ratio (OR) demonstrated in each study. The odds ratio (OR) was calculated from the data provided in the text in studies where it was not explicit in the text. A random-effects analysis model using the Review Manager software (RevMan 5.3, Copenhagen: The Nordic Cochrane Center, The Cochrane Collaboration, 2014) was used. Heterogeneity was assessed by calculating I^2^, and I^2^ > 60% was considered as high heterogeneity.

## 3. Results

### 3.1. Study Selection

**Figure 1:**
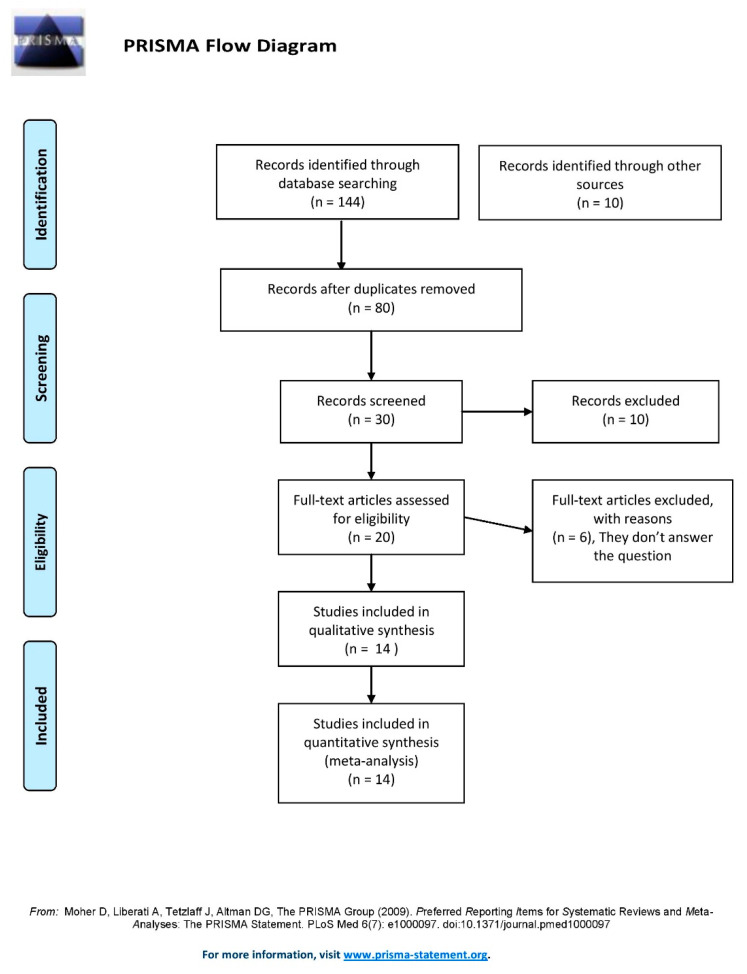
PRISMA chart for eligibility of studies obtained from the database search [15].

A total of one hundred and forty-four (n = 144) studies were identified with the search criteria mentioned. Ten (n = 10) additional studies were added from other sources. The duplicate citations were excluded and then study screening and selection were completed independently by two authors. The decision on differences among the reviewers was settled through mutual consensus or with the consultation of a third reviewer. After a full-text review of articles we found fourteen (n = 14) studies [1,2,3,4,5,6,7,8,9,10,11,12,13,14] eligible for this systematic review and meta-analysis. The study screening and selection process are shown as PRISMA flow diagram [15] in Figure 1.

### 3.2. Study Characteristics

All included studies were observational retrospective cohort studies. The studies included a total of 5185 patients with cSDH, of which 705 (13.6%) had recurrent cSDH and 4480 (86.4%) did not have recurrent cSDH. Motoei et al. [7] included the maximum number of patients (n = 787), of which 96 (12.2%) had recurrent cSDH and 691 (87.8%) did not have a recurrence. The study by Anderson et al. [1] had the maximum number of patients in the recurrence arm (n = 107), out of a total of 763 patients. The study by Hammer et al. [3] included 73 patients, of which 19 had recurrent cSDH (26%), the relative maximum among all the studies. Among all the studies an average of 50 patients (14.2%) patients of cSDH had a recurrence.. All the studies assessed risk factors for the development of recurrent cSDH as sex, mean age, symptoms, and cardiovascular disease. diabetes, hydrocephalus, stroke, alcohol, portal hypertension, hepatitis, use of anticoagulants, and trauma.

### 3.3. Study Quality

Eight (n = 8) studies [1,4,5,7,8,11,12,14] were graded as high quality and six (n = 6) studies [2,3,6,9,10,13] were graded as moderate quality as per Newcastle–Ottawa scale. Studies by Motoei et al. [7], Han et al. [4], and Kim et al. [5] had a score of 7/7, and studies by Torihashi et al. [12], Ohba et al. [8], You et al. [14], Anderson et al. [1], and Shen et al. [11] had a score of 6/7, suggesting high quality. Studies by Dos Santos et al. [10], Leroy et al. [6], and Chon et al. [2], each had a score of 5/7, and Oishi et al. [9], Yamamoto et al. [13], and Hammer et al. [3], each had a score of 4/7 suggestive of moderate study quality. Quality assessment of the included studies as per the Newcastle–Ottawa scale is shown in Table 1.

### 3.4. Outcome Measures

As described in the study methodology, we included all papers investigating the clinical and radiological risk factors associated with cSDH recurrence. We categorized the risk factors into the clinical and radiological parameters as described in the methodology section. The majority of the risk factors included in this study had a standardized method of assessment. The parameters extracted from the individual studies are shown in Table 2.

### 3.5. Results Synthesis and Meta-Analysis

#### 3.5.1. Clinical Risk Factors

Age >75 years was a significant risk factor for the development of recurrent cSDH with an odds ratio (OR) of 1.05 and a 95% confidence interval (CI) of 1.03–1.07. The studies in the meta-analysis for age as a risk factor had a high heterogeneity (I^2^ = 99%). This could be due to the non-significant relationship observed in Motoie et al. [7] and the largest number of patients among all the studies included for the quantitative synthesis. Forrest plot for age >75 years as a risk factor for the development of recurrent cSDH is as shown in Supplementary Figure S1. Data from the six (n = 6) studies [4,5,7,9,10,13] were extracted for meta-analysis for alcohol consumption as a predictor of the development of recurrent cSDH. None of the studies showed a significant relationship between alcohol and recurrent cSDH. Overall, there was no significant relationship between alcohol consumption and the development of recurrent cSDH with I^2^ at 29%. Data from twelve (n = 12) studies [1,2,4,5,6,7,8,9,11,12,13,14] were used to assess the role of anticoagulation of antiplatelet aggregation therapy in the development of recurrent cSDH. Anticoagulation therapy was found as a risk factor for recurrent cSDH in all the studies included in the meta-analysis except the study by Anderson et al. [1]. The fixed effect model was used in the synthesis of the results as there was low heterogeneity I^2^ of 38% in the studies. Overall, the risk factor of 1.5 in favor of anticoagulation or anti-aggregation therapy was noticed after the quantitative synthesis with OR 1.28 CI (1.02–1.62). The presence of chronic kidney disease had a risk factor of 1.8 for the development of recurrence, yet it was not found to be a significant risk factor as per studies by Han et al. [4], and Kim et al. [5]. Six studies [1,2,4,10,11,12] found a high risk of development of cSDH in presence of diabetes mellitus. Diabetes mellitus was found to be a significant risk factor for the development of recurrent cSDH with a risk factor of 1.4, OR 1.53, 95% CI (1.24–1.90). Likewise, liver disease, obesity, and seizures were significant risk factors for the development of recurrence in cSDH. On the other hand, gender, trauma, heart disease, malignancy, smoking, stroke, and hypertension was not found to be significant risk factors. Forrest plots depicting the significance of clinical risk factors are shown in Supplementary Figures S1–S15.

#### 3.5.2. Radiological Risk Factors

The bilateral hematoma was found to be a significant predictor of cSDH recurrence. In addition, the internal architecture of cSDH also played a significant role in the recurrence of cSDH. The more a cSDH is heterogeneous in CT density, the more likely it is to develop recurrence. The laminar internal architecture was in favor of the development of recurrence as compared to the isodense, hypodense, or hyperdense cSDH. Studies reported that separated cSDH has a higher incidence of recurrence as compared with trabeculated appearance. We found a similar association in our meta-analysis. Additionally, the width of the hematoma correlated positively with the development of recurrence in cSDH. Interestingly the studies comparing the impact of midline shift on the development of recurrence reported a greater risk of recurrence with greater midline shift. After meta-analysis, midline shift was found to be a significant factor defining the recurrence of cSDH with OR 1.75, 95% CI (1.49–2.07). As expected, and further supported by many studies, pneumocephalus was a strong and significant predictor for the development of recurrence in cSDH with OR 2.36, 95% CI (1.41–3.96). Forrest plots depicting the significance of radiological risk factors are shown in Supplementary Figures S16–S27.

The odd’s ratio of the risk factor from the pooled analysis of the included studies is presented in Table 3.

## 4. Discussion

Recurrence after adequate surgical management of cSDH has been abundantly reported in the literature, with rates varying between 8% and 39%, with an average of 16% [16,17,18,19,20]. Even though the literature has many systematic reviews comparing results of management strategies in cSDH, to the best of our knowledge, to date there has been only one systematic review on implicated clinical and radiological factors which may be predictors of recurrence of cSDH [21]. Even though this article is titled to be a systematic review, as per standards, it does not qualify for the same as it does not answer specific questions based on statistical analysis. There are several individual institutional studies in the literature that have tried to analyze these factors as a determinant of recurrent chronic subdural hematomas.

### 4.1. Age

It has been consistently reported in the literature that age is not a predictor of cSDH recurrence. Motoie et al. found that age was not a significant predictor of cSDH recurrence when considering 75 as the cut-off age in the largest retrospective analysis of 787 patients carried out in 2018. [7]. In the present meta-analysis, we found that the age of fewer than 75 years is a predictor of cSDH recurrence. However, caution should still be exercised because of the heterogeneity of the data, which indicates that there is a very high likelihood that it is not simply a matter of coincidence.

### 4.2. Gender

Various studies have provided mixed results regarding the predictability of male sex as a risk factor for cSDH recurrence [2,4,5,6,7,8,9,10,11,12,13,14]. While the largest of the studies by Han et al. (2017) and Motoie et al. (2018) did show that the male gender is predictive, the results were not significant. In the pooled estimate, we found that there was no effect on gender cSDH recurrence. (Figure S6). The heterogeneity for the male sex as a predictor of recurrence was low at 32%.

### 4.3. Co-Morbidities: Hypertension, Diabetes, and Obesity

The predictive value of concurrent hypertension was not predictive of recurrent cSDH. Conversely, it is more certain to say that diabetes and obesity are predictors of recurrence. Angiogenesis and capillary vasculopathy might play a role in the development of recurrence. It is well-known that the membranes of cSDH have a network of fragile capillaries that are responsible for the growth and recurrence of cSDH. Diabetes mellitus harms the smaller blood vessels as evidenced by the systemic effects; for example, vasculopathy in other organ systems. Accordingly, DM likely induced capillary vasculopathy might be responsible for consistent effect in an increased risk of recurrence in most of the studies. The studies which did not find an association between the DM and risk of recurrence propose that DM increases blood viscosity through the changes in osmotic pressure and thereby induces coagulation and decreases the risk of hematoma. Alternatively, DM could be an independent risk factor for the recurrence of hematoma. In the present meta-analysis, we find that hypertension did not affect the cSDH recurrence. This could be due to the angiotensin-converting enzyme inhibitors frequently taken by hypertensives [22]. An increase in cSDH is regulated by a complex array of factors including activated blood products and growth factors mediating fibrinolysis. Therefore, future studies on vasculopathy in cSDH will shed more light on these effects.

### 4.4. Anticoagulation and Anti-Aggregation Therapy

Patients with cSDH are often elderly patients who are either on anticoagulation or antiplatelet therapy and may occasionally be associated with a history of trivial trauma. In clinical practice, these factors in combination are considered to predispose the chances of having subdural bleeding in the first place. It is currently debatable if they can lead to cSDH recurrence after receiving adequate surgical treatment. [1,2,4,5,6,7,8,9,12,13,14]. However, with acceptable heterogeneity and statistical significance, both anticoagulant use and antiplatelet administration are predictive of recurrence of cSDH.

### 4.5. Concomitant Stroke, Malignancy, and Chronic Heart, Kidney, and Liver Diseases

Among all the above conditions, only liver disease had a positive correlation with recurrent cSDHs which was statistically significant. All the other conditions remain non-predictive.

### 4.6. Trauma, Alcohol Consumption, and Smoking Habits

As expected, a history of trauma, as well as alcohol and smoking, are unrelated and do not predispose to the recurrence of chronic SDH.

### 4.7. Midline Shift > 10 mm and Hematoma Width >20 mm on Imaging

All studies showed that a midline shift of >10 mm and/or a hematoma width of >20 mm have a strong predictive value which is statistically significant.

### 4.8. Hematoma Characteristics

The only positive predictor which significantly increased the risk of cSDH recurrence was a septate hematoma. The remaining hematoma subtypes and densities did not affect the chances of cSDH recurrence after adequate evacuation.

### 4.9. Bilaterality, Severe Brain Atrophy, and Postoperative Pneumocephalus

Bilaterality may have minimal risk of cSDH recurrence, and severe brain atrophy and postoperative pneumocephalus are definitive and significant predisposing factors for recurrent cSDH formation.

### 4.10. Implications

We identified several clinical and radiological factors as predictors of cSDH recurrence. Since many times recurrent SDH patients must first become symptomatic before receiving therapy, it is challenging to estimate how many patients genuinely have the condition. Additionally, persistent bleeding might be fatal in certain patients. Therefore, identification of these risk factors will help in identifying patients at risk of recurrence and can be followed up more frequently. These clinical and radiological parameters should be validated further in larger multicentric studies thereby enabling guidelines and recommendations for the management of cSDH to decrease the recurrence. As all the studies included in the present review were retrospective in nature, the strength of evidence obtained is very low and cannot guide recommendations at the present stage. In some of the variables assessed there was high heterogeneity due to the study designs and participants and this also affects the strength of the evidence available.

Middle meningeal artery embolization has been recently described as an alternative and minimally invasive treatment for recurrent cSDH and prophylaxis treatment at high-risk patients. Therefore, if the factors that predict recurrence are identified with high-quality evidence, patients can be counseled for emerging therapies such as middle meningeal artery embolization.

## 5. Conclusions

Age >75 years, use of anticoagulation therapy, liver disease, and obesity were significant risk factors for the development of cSDH recurrence. Pneumocephalus, internal architecture of hematoma, bilateral cSDH, the width of hematoma, and the presence of bilateral cSDH are important radiological parameters of the development of recurrent cSDH.

## Figures and Tables

**Table 1:** Newcastle–Ottawa scale for quality assessment of the fourteen qualified studies included in this meta-analysis.

Study	Representativeness of Sample	Size Sample	Source of Information	Demonstration That Outcome Was Not Present at Study Start	Confusion Variable Control	Assessment of Outcome	Enough Follow-Up Period
**Oishi et al. 2001**			**★**	**★**		**★**	**★**
**Yamamoto et al. 2003**	**★**		**★**	**★**		**★**	**★**
**Torihashi et al. 2008**	**★**	**★**	**★**	**★**	**★**	**★**	
**Chon et al. 2012**	**★**	**★**	**★**	**★**	**★**		
**Ohba et al. 2012**	**★**	**★**	**★**		**★**	**★**	**★**
**Leroy et al. 2015**	**★**		**★**	**★**	**★**	**★**	
**Kim et al. 2015**	**★**	**★**	**★**	**★**	**★**	**★**	**★**
**Han et al. 2016**	**★**	**★**	**★**	**★**	**★**	**★**	**★**
**Hammer et al. 2017**	**★**	**★**	**★**	**★**			
**Motoie et al. 2018**	**★**	**★**	**★**	**★**	**★**	**★**	**★**
**You et al. 2018**	**★**	**★**	**★**	**★**	**★**	**★**	
**Andersen et al. 2019**	**★**	**★**	**★**	**★**		**★**	**★**
**Dos Santos et al. 2019**			**★**	**★**	**★**	**★**	**★**
**Shen et al. 2019**	**★**	**★**	**★**	**★**	**★**	**★**	

★ Indicates that it meets criteria in Newcastle–Ottawa Scale.

**Table 2:** Summary of the 14 qualified studies used in this meta-analysis.

Study	Year	Type	Subgroup	Clinical Risk Factors	Risk Factor in CT	Newcastle–Ottawa Scale Scoring
**Oishi et al.**	2001	Observational retrospective cohort	Recurrence group (n = 10) No recurrent group (n = 106)	Sex (male and female) Mean age (years old) Alcohol abuse Antiaggregant or anticoagulant History of head injury Days from appearance Symptoms	Hematoma density (low, iso, high, normal, mixed) Brain atrophy (no or mild atrophy, definite atrophy and severe atrophy) Amount of residual air (no or mild, definite and severe atrophy)	4/7
**Yamamoto et al.**	2003	Observational retrospective cohort	Recurrence group (n = 11) No recurrence group (n = 94)	Sex (male and female) Mean age (years old) Trauma (present, absent) Symptoms (present, absent) Medical history: seizure, smoking, hypertension, diabetes, cerebrovascular disease, heart and liver disease, alcohol abuse, antiaggregant or anticoagulant drugs	Hematoma site Layering of hematoma Multiplicity of hematoma cavity Development of capsule Midline shift Hematoma width (mm) Intracranial air (7 days postop)	4/7
**Torihashi et al.**	2008	Observational retrospective cohort	Recurrence group (n = 61) No recurrence group (n = 276)	Sex (male and female) Mean age (years old) Odds ratio to: diabetes mellitus, hypertension, atrial fibrillation, heart disease, antiaggregant or anticoagulant drugs, cerebrovascular disease, and drainage tube placement	Bilateral hematoma	6/7
**Chon et al.**	2012	Observational retrospective cohort	Recurrence group (n = 92) No recurrence group (n = 328)	Sex (male and female) Mean age (years old) medical history: seizure, smoking, hypertension, diabetes, cerebrovascular disease, heart and liver disease, alcohol abuse, antiaggregant or anticoagulant drugs, and head trauma	Hematoma location (left, right or bilateral) Width of hematoma (≥20 mm or ≤20 mm) Hematoma density ( low, iso, high, normal, mixed) Internal architecture hematoma (homogeneous, laminar, separated or trabeculated) Brain atrophy (grades 0–1, 2, 3) Preoperative midline shift	5/7
**Ohba et al.**	2012	Observational retrospective cohort	Recurrence group (n = 20) No recurrence group (n = 117)	Odds ratio to: age (>70 years, <70 years), sex (male) antiaggregant or anticoagulant drugs	Hematoma location (left) Width of hematoma (≥20 mm) Internal architecture hematoma (homogeneous, laminar, separated or trabecula) Midline shift (>10 mm) Direction (anterior) Massive air collection,	6/7
**Leroy et al.**	2015	Observational retrospective cohort	Recurrence group (n = 24) No recurrence group (n = 116)	Sex (male and female) Mean age (years old) Medical history: antiaggregant or anticoagulant drugs; head trauma; SeizuresPreoperative GCS < 15; craniotomy	Homogeneous hematoma Midline shift Hematoma location (right, left side, bilateral) Residual hematoma thickness.	5/7
**Kim et al.**	2015	Observational retrospective cohort	Recurrence group (n = 31) No recurrence group (n = 337)	Sex (male and female) Mean age (years old) Symptoms Medical history: seizure; hypertension, diabetes; anticoagulant drugs; malignancy; chronic renal disease	Hematoma type: type I (single, double and multiple layer); and type II (isodensity, hypodensity and mixed) Hematoma laterality: (left/right)-, uni or bilateral	7/7
**Han et al.**	2016	Observational retrospective cohort	Recurrence group (n = 104) No recurrence group (n = 652)	Sex (male and female) Age (>75 and <75 years old) BMI: ≥25.0 kg/m^2^ (obesity) Medical history: seizure; hypertension, diabetes; anticoagulant drugs; malignancy; chronic renal and liver disease; heart disease; cerebrovascular disease; alcohol; smoking	Hematoma width >20 mm Midline shift >10 mm Hematoma laterality: unilateral or bilateral	7/7
**Hammer et al.**	2017	Observational retrospective cohort	Recurrence group (n = 19) No recurrence group (n = 54)	Presence or absence of epilepsy Symptoms	Hematoma density (low, iso, high, normal, mixed) Internal architecture of the hematoma (homogeneous, laminar, separated or trabeculated)	4/7
**Motoie et al.**	2018	Observational retrospective cohort	Recurrence group (n = 96) No recurrence group (n = 691)	Sex (male or female) Age >75 years Medical history: smoking, hypertension, diabetes, stroke, heart and liver disease, alcohol abuse, antiaggregant or anticoagulant drugs, cancer	Bilateral hematoma	7/7
**You et al.**	2018	Observational retrospective cohort	Recurrence group (n = 34) No recurrence group (n = 192)	Age, mean ± SD (years) Sex (male and female) BMI, mean GCS at admission, median (IQR) Medical history: head trauma; antiaggregant or anticoagulant drugs; hypertension, diabetes, heart disease.	Hematoma location (left, right or bilateral) Hematoma density (low, iso, high, normal, mixed) Internal architecture of the hematoma (homogeneous, laminar, separated or trabeculated)	6/7
**Andersen et al.**	2019	Observational retrospective cohort	Recurrence group (n = 107) No recurrence group (n = 656)	Sex (male and female) Mean age (years old) Symptoms Medical history: hypertension, diabetes; anticoagulant drugs; previous craniotomy; head trauma Glasgow come scale	Hematoma location (left, right or bilateral) Hematoma density (low, iso, high, normal, mixed) Internal architecture hematoma (homogeneous, laminar, separated or trabeculated)	6/7
**Dos Santos et al.**	2019	Observational retrospective cohort	Recurrence group (n = 27) No recurrence group (n = 473)	Sex (male and female) Mean age (years old) Symptoms Medical history: hypertension, diabetes; hydrocephalus, stroke, prematurity, alcohol, portal hypertension, hepatitis, atrial fibrillation	Bilateral hematoma	5/7
**Shen et al.**	2019	Observational retrospective cohort	Recurrence group (n = 69) No recurrence group (n = 388)	Sex (male and female) Age(>75 and <75 years old) Medical history: hypertension, diabetes; anticoagulant or antiaggregant drugs; cerebrovascular disease Glasgow come scale	Unilateral or bilateral Hematoma side Midline shift (>10 mm) Brain atrophy (no or mild atrophy, definite atrophy and severe atrophy) Hematoma density (low, iso, high, normal, mixed) Internal architecture hematoma (homogeneous, laminar, separated or trabeculated)	6/7

**Table 3:** Odd’s ratio of risk factors of recurrence of chronic subdural hematoma.

S. No.	Risk Factor	Odd’s Ratio (95% Confidence Interval)	*p*-Value
1.	Age > 75 years	1.05 (1.03–1.07)	*p* < 0.00001
**2.**	**Alcohol consumption**	**1.10 (0.81–1.48)**	***p* = 0.55**
3.	Anticoagulation or anti-aggregation therapy	1.28 (1.02–1.62)	*p* = 0.03
**4.**	**Chronic kidney disease**	**1.21 (0.63–2.35)**	***p* = 0.56**
5.	Diabetes mellitus	1.53 (1.24–1.90)	*p* < 0.0001
**6.**	**Gender male**	**1.20 (0.96–1.49)**	***p* = 0.10**
**7.**	**Trauma**	**0.94 (0.71–1.24)**	***p* = 0.65**
**8.**	**Heart Diseases**	**1.23 (0.83–1.84)**	***p* = 0.31**
**9.**	**Hypertension**	**1.12 (0.95–1.32)**	***p* = 0.18**
10.	Liver disease	1.83 (1.23–2.73)	*p* = 0.003
**11.**	**Malignancy**	**1.10 (0.70–1.72)**	***p* = 0.67**
12.	Obesity	1.81 (1.09–3.01)	*p* = 0.02
13.	Seizure	2.78 (1.57–4.92)	*p* = 0.0004
**14.**	**Smoking**	**1.03 (0.74–1.42)**	***p* = 0.87**
**15.**	**Stroke**	**1.25 (0.88–1.77)**	***p* = 0.21**
16.	Bilateral hematoma	1.31 (1.05–1.63)	*p* = 0.02
17.	Severe brain atrophy	2.61 (1.88–3.64)	*p* < 0.00001
18.	Internal architecture of the hematoma: homogeneous	1.42 (1.07–1.88)	*p* = 0.01
**19.**	**Hematoma hyperdensity**	**1.33 (0.81–2.17)**	***p* = 0.26**
**20.**	**Hematoma hypodensity**	**0.86 (0.56–1.34)**	***p* = 0.51**
**21.**	**Hematoma isodensity**	**0.78 (0.57–1.07)**	***p* = 0.12**
22.	Internal architecture of the hematoma: laminar	1.57 (1.03–2.39)	*p* = 0.04
23.	Midline shift > 10 mm	1.75 (1.49–2.07)	*p* < 0.00001
24.	Postoperative pneumocephalus	2.36 (1.41–3.96)	*p* = 0.001
25.	Internal architecture of the hematoma: separated	2.33 (1.69–3.19)	*p* < 0.00001
**26.**	**Internal architecture of the hematoma: trabeculate**	**0.89 (0.55–1.42)**	***p* = 0.61**
27.	Width of hematoma > 20 mm	1.22 (1.05–1.41)	*p* = 0.007

## Supplementary Materials

The following supporting information can be downloaded at: https://www.mdpi.com/article/10.3390/neurolint14030057/s1, Figure S1–S27.

## References

[1] Andersen-Ranberg N.C., Debrabant B., Poulsen F.R., Bergholt B., Hundsholt T., Fugleholm K. (2019). The Danish chronic subdural hematoma study-predicting recurrence of chronic subdural hematoma. Acta Neurochir..

[2] Chon K.H., Lee J.M., Koh E.J., Choi H.Y. (2012). Independent predictors for recurrence of chronic subdural hematoma. Acta Neurochir..

[3] Hammer A., Tregubow A., Kerry G., Schrey M., Hammer C., Steiner H.-H. (2017). Predictors for Recurrence of Chronic Subdural Hematoma. Turk. Neurosurg..

[4] Han M.-H., Ryu J.I., Kim C.H., Kim J.M., Cheong J.H., Yi H.-J. (2017). Predictive factors for recurrence and clinical outcomes in patients with chronic subdural hematoma. J. Neurosurg..

[5] Kim J., Moon J., Kim T., Ahn S., Hwang G., Bang J., Kwon O.-K., Oh C.W. (2015). Risk Factor Analysis for the Recurrence of Chronic Subdural Hematoma: A Review of 368 Consecutive Surgical Cases. Korean J. Neurotrauma.

[6] Leroy H.-A., Aboukaïs R., Reyns N., Bourgeois P., Labreuche J., Duhamel A., Lejeune J.-P. (2015). Predictors of functional outcomes and recurrence of chronic subdural hematomas. J. Clin. Neurosci..

[7] Motoie R., Karashima S., Otsuji R., Ren N., Nagaoka S., Maeda K., Ikai Y., Uno J., Gi H. (2018). Recurrence in 787 Patients with Chronic Subdural Hematoma: Retrospective Cohort Investigation of Associated Factors Including Direct Oral Anticoagulant Use. World Neurosurg..

[8] Ohba S., Kinoshita Y., Nakagawa T., Murakami H. (2013). The risk factors for recurrence of chronic subdural hematoma. Neurosurg. Rev..

[9] Oishi M., Toyama M., Tamatani S., Kitazawa T., Saito M. (2001). Clinical factors of recurrent chronic subdural hematoma. Neurol. Med.-Chir..

[10] dos Santos R.G., Xander P.A.W., Rodrigues L.H.D.S., da Costa G.H.F., Veiga J.C.E., de Aguiar G.B. (2019). Analysis of predisposing factors for chronic subdural hematoma recurrence. Rev. Assoc. Med. Bras..

[11] Shen J., Yuan L., Ge R., Wang Q., Zhou W., Jiang X.C., Shao X. (2019). Clinical and radiological factors predicting recurrence of chronic subdural hematoma: A retrospective cohort study. Injury.

[12] Torihashi K., Sadamasa N., Yoshida K., Narumi O., Chin M., Yamagata S. (2008). Independent predictors for recurrence of chronic subdural hematoma: A review of 343 consecutive surgical cases. Neurosurgery.

[13] Yamamoto H., Hirashima Y., Hamada H., Hayashi N., Origasa H., Endo S. (2003). Independent predictors of recurrence of chronic subdural hematoma: Results of multivariate analysis performed using a logistic regression model. J. Neurosurg..

[14] You W., Zhu Y., Wang Y., Liu W., Wang H., Wen L., Yang X. (2018). Prevalence of and risk factors for recurrence of chronic subdural hematoma. Acta Neurochir..

[15] Moher D., Liberati A., Tetzlaff J., Altman D.G., PRISMA Group (2009). Preferred reporting items for systematic reviews and meta-analyses: The PRISMA statement. PLoS Med..

[16] Spallone A., Giuffre R., Gagliardi F.M., Vagnozzi R. (1989). Chronic subdural hematoma in extremely aged patients. Eur. Neurol..

[17] Mori K., Maeda M. (2001). Surgical treatment of chronic subdural hematoma in 500 consecutive cases: Clinical characteristics, surgical outcome, complications, and recurrence rate. Neurol. Med. -Chir..

[18] Lind C.R., Lind C.J., Mee E.W. (2003). Reduction in the number of repeated operations for the treatment of subacute and chronic subdural hematomas by placement of subdural drains. J. Neurosurg..

[19] Abouzari M., Rashidi A., Rezaii J., Esfandiari K., Asadollahi M., Aleali H., Abdollahzadeh M. (2007). The role of postoperative patient posture in the recurrence of traumatic chronic subdural hematoma after burr-hole surgery. Neurosurgery.

[20] Ko B.-S., Lee J.-K., Seo B.-R., Moon S.-J., Kim J.-H., Kim S.-H. (2008). Clinical analysis of risk factors related to recurrent chronic subdural hematoma. J. Korean Neurosurg. Soc..

[21] Abdelsadg M., Kanodia A.K., Abbas A., Sheikh A. (2017). Chronic subdural haematoma: Systematic review highlighting risk factors for recurrent bleeds. Neuro. Open J..

[22] Weigel R., Hohenstein A., Schlickum L., Weiss C., Schilling L. (2007). Angiotensin converting enzyme inhibition for arterial hypertension reduces the risk of recurrence in patients with chronic subdural hematoma possibly by an antiangiogenic mechanism. Neurosurgery.

