# Supplementary material for: Clinical and Radiological Characteristics for Recurrence of Chronic Subdural Hematoma: A Systematic Review and Meta-Analysis
Source: Neurol Int. 2022 Aug 26;14(3):683–95. doi: 10.3390/neurolint14030057 (PMC9505137; doi:10.3390/neurolint14030057)
Supplement: Supplementary file 1 [file neurolint-14-00057-s001.zip › neurolint-1843509-supplementary.pdf]

Figure S1. Forest plot comparison for risk of chronic subdural hematoma recurrence: Clinical History, Risk factor: 1.1 Age >75 Years.

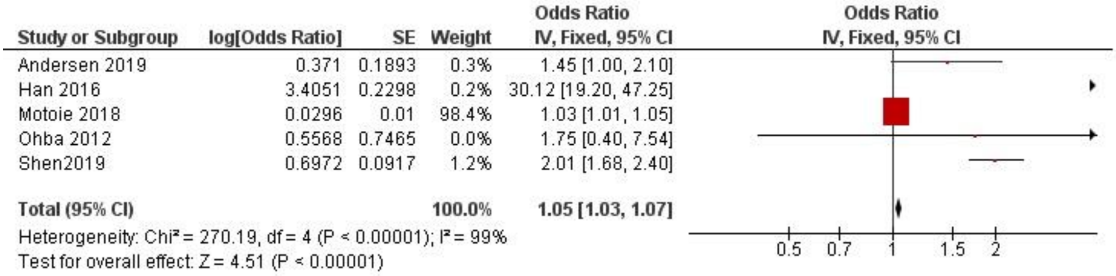

Study or Subgroup	log[Odds Ratio]	SE	Weight	Odds Ratio IV, Fixed, 95% CI
Andersen 2019	0.371	0.1893	0.3%	1.45 [1.00, 2.10]
Han 2016	3.4051	0.2298	0.2%	30.12 [19.20, 47.25]
Motoie 2018	0.0296	0.01	98.4%	1.03 [1.01, 1.05]
Ohba 2012	0.5568	0.7465	0.0%	1.75 [0.40, 7.54]
Shen2019	0.6972	0.0917	1.2%	2.01 [1.68, 2.40]
**Total (95% CI)**			**100.0%**	**1.05 [1.03, 1.07]**

Heterogeneity: Chi² = 270.19, df = 4 (P < 0.00001); I² = 99%
Test for overall effect: Z = 4.51 (P < 0.00001)

Forest plot of comparison: 1 Risk factor of Chronic Subdural Hematoma recurrence: Clinical History, Risk factor: 1.1 Age (>75 years old).

Figure S2. Forest plot comparison for risk of chronic subdural hematoma recurrence, Risk factor: 1.7 alcohol consumption.

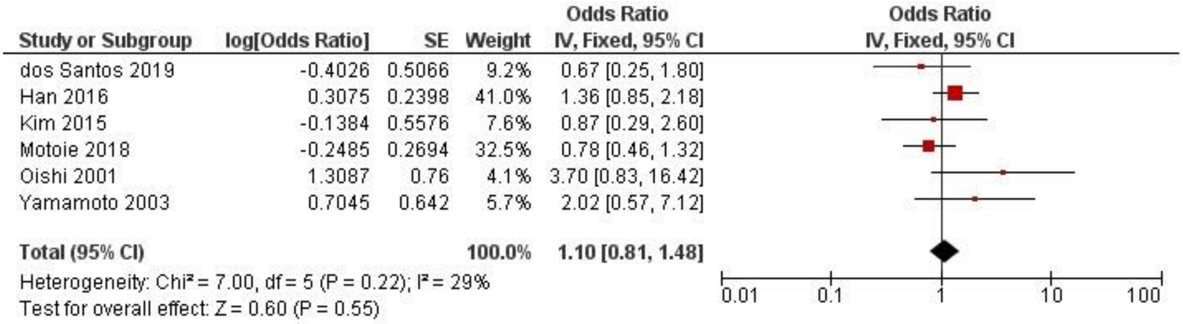

Study or Subgroup	log[Odds Ratio]	SE	Weight	Odds Ratio IV, Fixed, 95% CI
dos Santos 2019	-0.4026	0.5066	9.2%	0.67 [0.25, 1.80]
Han 2016	0.3075	0.2398	41.0%	1.36 [0.85, 2.18]
Kim 2015	-0.1384	0.5576	7.6%	0.87 [0.29, 2.60]
Motoie 2018	-0.2485	0.2694	32.5%	0.78 [0.46, 1.32]
Oishi 2001	1.3087	0.76	4.1%	3.70 [0.83, 16.42]
Yamamoto 2003	0.7045	0.642	5.7%	2.02 [0.57, 7.12]
**Total (95% CI)**			**100.0%**	**1.10 [0.81, 1.48]**

Heterogeneity: Chi² = 7.00, df = 5 (P = 0.22); I² = 29%
Test for overall effect: Z = 0.60 (P = 0.55)

Forest plot of comparison: 1 Risk factor of Chronic Subdural Hematoma recurrence, Risk factor : 1.7 alcohol consumption.

Figure S3. Forest plot comparison for risk of chronic subdural hematoma recurrence, Risk factor: 1.5 Anticoagulation or antiagregation therapy.

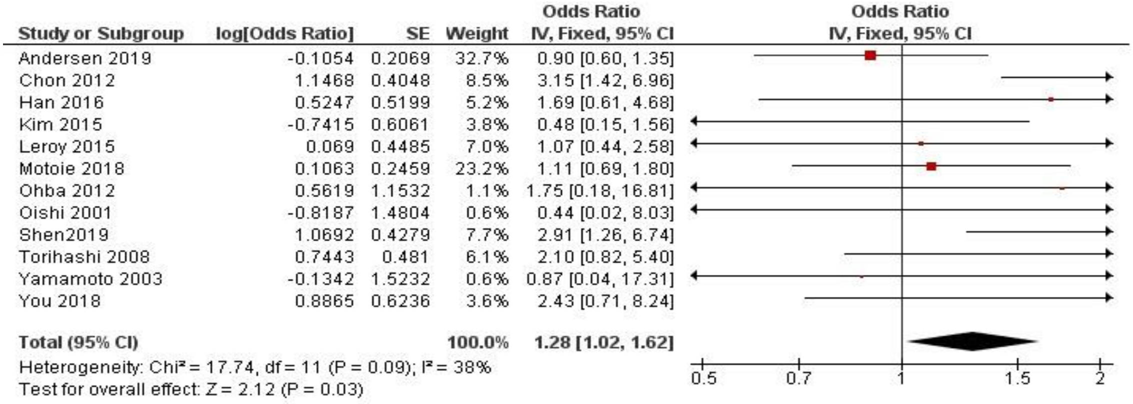

Study or Subgroup	log[Odds Ratio]	SE	Weight	Odds Ratio IV, Fixed, 95% CI
Andersen 2019	-0.1054	0.2069	32.7%	0.90 [0.60, 1.35]
Chon 2012	1.1468	0.4048	8.5%	3.15 [1.42, 6.96]
Han 2016	0.5247	0.5199	5.2%	1.69 [0.61, 4.68]
Kim 2015	-0.7415	0.6061	3.8%	0.48 [0.15, 1.56]
Leroy 2015	0.069	0.4485	7.0%	1.07 [0.44, 2.58]
Motoie 2018	0.1063	0.2459	23.2%	1.11 [0.69, 1.80]
Ohba 2012	0.5619	1.1532	1.1%	1.75 [0.18, 16.81]
Oishi 2001	-0.8187	1.4804	0.6%	0.44 [0.02, 8.03]
Shen2019	1.0692	0.4279	7.7%	2.91 [1.26, 6.74]
Torihashi 2008	0.7443	0.481	6.1%	2.10 [0.82, 5.40]
Yamamoto 2003	-0.1342	1.5232	0.6%	0.87 [0.04, 17.31]
You 2018	0.8865	0.6236	3.6%	2.43 [0.71, 8.24]
**Total (95% CI)**			**100.0%**	**1.28 [1.02, 1.62]**

Heterogeneity: Chi² = 17.74, df = 11 (P = 0.09); I² = 38%
Test for overall effect: Z = 2.12 (P = 0.03)

Forest plot of comparison: 1 Risk factor of Chronic Subdural Hematoma recurrence, Risk factor: 1.5 Anticoagulation or antiagregation therapy.

Figure S4. Forest plot comparison for risk of chronic subdural hematoma recurrence: Clinical History, Risk factor: 1.8 Chronic kidney disease.

Study or Subgroup	log[Odds Ratio]	SE	Weight	Odds Ratio IV, Fixed, 95% CI
Han 2016	0.1823	0.3452	95.1%	1.20 [0.61, 2.36]
Kim 2015	0.4167	1.5233	4.9%	1.52 [0.08, 30.03]
**Total (95% CI)**			**100.0%**	**1.21 [0.63, 2.35]**

Heterogeneity: $Chi^2 = 0.02$, df = 1 (P = 0.88); $I^2 = 0\%$
Test for overall effect: Z = 0.58 (P = 0.56)

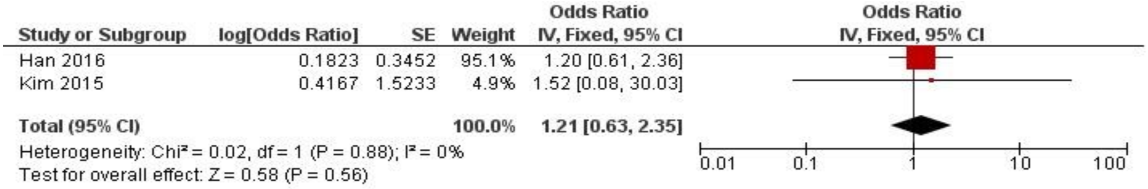

Forest plot of comparison: 1 Risk factor of Chronic Subdural Hematoma recurrence: Clinical History, Risk factor: 1.8 Chronic kidney disease.

Figure S5. Forest plot comparison for risk of chronic subdural hematoma recurrence, Risk factor: 1.4 Diabetes Mellitus.

Study or Subgroup	log[Odds Ratio]	SE	Weight	Odds Ratio IV, Fixed, 95% CI
Andersen 2019	0.2624	0.2477	19.4%	1.30 [0.80, 2.11]
Chon 2012	0.8566	0.2801	15.2%	2.36 [1.36, 4.08]
dos Santos 2019	2.0272	0.5826	3.5%	7.59 [2.42, 23.79]
Han 2016	0.3221	0.2069	27.8%	1.38 [0.92, 2.07]
Kim 2015	0.0625	0.4758	5.3%	1.06 [0.42, 2.70]
Motoie 2018	-0.0202	0.2765	15.6%	0.98 [0.57, 1.68]
Shen2019	0.8181	0.3959	7.6%	2.27 [1.04, 4.92]
Torihashi 2008	1.0578	0.5767	3.6%	2.88 [0.93, 8.92]
Yamamoto 2003	-0.8622	1.0811	1.0%	0.42 [0.05, 3.51]
You 2018	-0.4842	1.0709	1.0%	0.62 [0.08, 5.03]
**Total (95% CI)**			**100.0%**	**1.53 [1.24, 1.90]**

Heterogeneity: $Chi^2 = 18.12$, df = 9 (P = 0.03); $I^2 = 50\%$
Test for overall effect: Z = 3.92 (P < 0.0001)

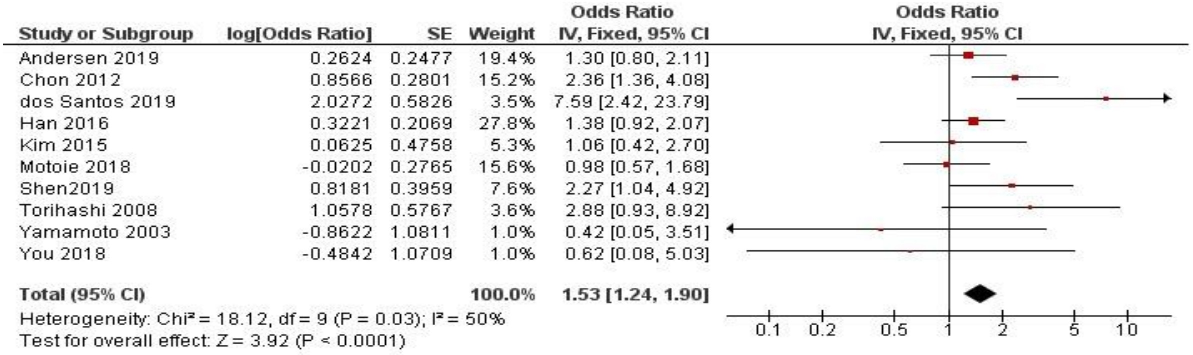

Forest plot of comparison: 1 Risk factor of Chronic Subdural Hematoma recurrence, Risk factor: 1.4 Diabetes Mellitus.

Figure S6. Forest plot comparison for risk factor of chronic subdural hematoma recurrence: Clinical history, Risk factor: 1.2 Gender (Male).

Study or Subgroup	log[Odds Ratio]	SE	Weight	Odds Ratio IV, Fixed, 95% CI
Chon 2012	-0.2596	0.2813	15.5%	0.77 [0.44, 1.34]
dos Santos 2019	-0.3567	0.4323	6.6%	0.70 [0.30, 1.63]
Han 2016	0.2624	0.2477	20.0%	1.30 [0.80, 2.11]
Kim 2015	1.269	0.6196	3.2%	3.56 [1.06, 11.98]
Leroy 2015	0.9151	0.5373	4.3%	2.50 [0.87, 7.16]
Motoie 2018	0.5653	0.2684	17.0%	1.76 [1.04, 2.98]
Ohba 2012	0.9207	0.6416	3.0%	2.51 [0.71, 8.83]
Oishi 2001	0.4568	0.8194	1.8%	1.58 [0.32, 7.87]
Shen2019	-0.0884	0.3356	10.9%	0.92 [0.47, 1.77]
Torihashi 2008	-0.3425	0.3298	11.3%	0.71 [0.37, 1.36]
Yamamoto 2003	0.7464	0.8124	1.9%	2.11 [0.43, 10.37]
You 2018	0.3254	0.5176	4.6%	1.38 [0.50, 3.82]
**Total (95% CI)**			**100.0%**	**1.20 [0.96, 1.49]**

Heterogeneity: $Chi^2 = 16.28$, df = 11 (P = 0.13); $I^2 = 32\%$
Test for overall effect: Z = 1.63 (P = 0.10)

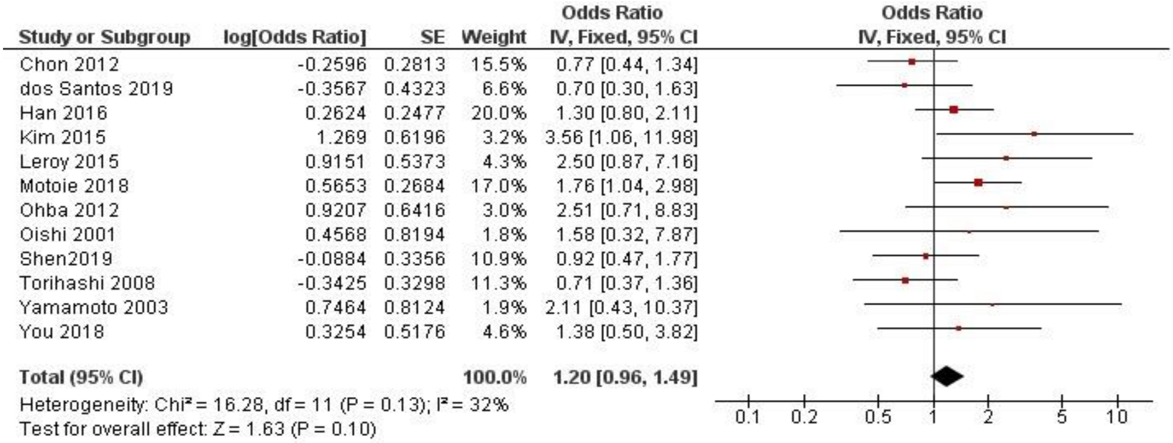

Forest plot of comparison: 1 Risk factor of Chronic Subdural Hematoma recurrence: Clinical History, Risk factor: 1.2 Gender (Male).

Figure S7. Forest plot comparison for risk factor of chronic subdural hematoma recurrence: Clinical history, Risk factor: 1.15 trauma.

Study or Subgroup	log[Odds Ratio]	SE	Weight	Odds Ratio IV, Fixed, 95% CI
Andersen 2019	-0.1054	0.2069	47.6%	0.90 [0.60, 1.35]
Chon 2012	0.0616	0.2385	35.8%	1.06 [0.67, 1.70]
Leroy 2015	0.091	0.4624	9.5%	1.10 [0.44, 2.71]
Oishi 2001	-1.5275	0.7175	4.0%	0.22 [0.05, 0.89]
Yamamoto 2003	0.4888	0.8158	3.1%	1.63 [0.33, 8.07]
**Total (95% CI)**			**100.0%**	**0.94 [0.71, 1.24]**

Heterogeneity: Chi² = 5.05, df = 4 (P = 0.28); I² = 21%
Test for overall effect: Z = 0.45 (P = 0.65)

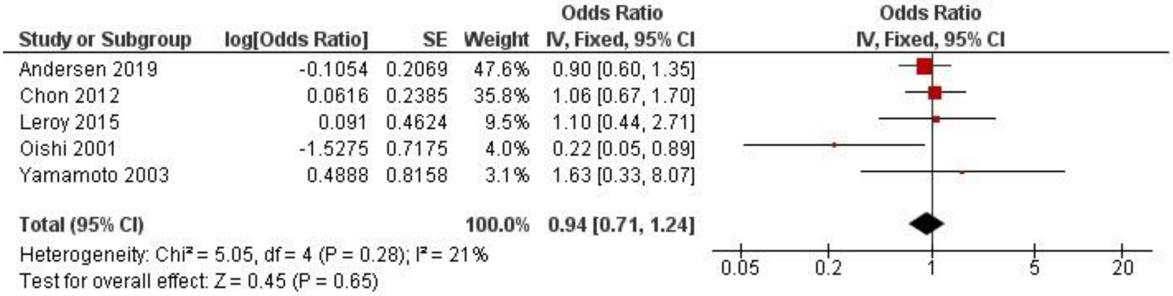

Forest plot of comparison: 1 Risk factor of Chronic Subdural Hematoma recurrence: Clinical History, Risk factor : 1.15 Head trauma.

Figure S8. Forest plot comparison for risk factor of chronic subdural hematoma recurrence: Clinical history, Risk factor: 1.10 Heart diseases

Study or Subgroup	log[Odds Ratio]	SE	Weight	Odds Ratio IV, Fixed, 95% CI
Han 2016	0.1133	0.2471	68.6%	1.12 [0.69, 1.82]
Torihashi 2008	0.1873	0.4452	21.1%	1.21 [0.50, 2.89]
Yamamoto 2003	-0.2816	1.0968	3.5%	0.75 [0.09, 6.48]
You 2018	1.5148	0.788	6.7%	4.55 [0.97, 21.31]
**Total (95% CI)**			**100.0%**	**1.23 [0.83, 1.84]**

Heterogeneity: Chi² = 3.10, df = 3 (P = 0.38); I² = 3%
Test for overall effect: Z = 1.02 (P = 0.31)

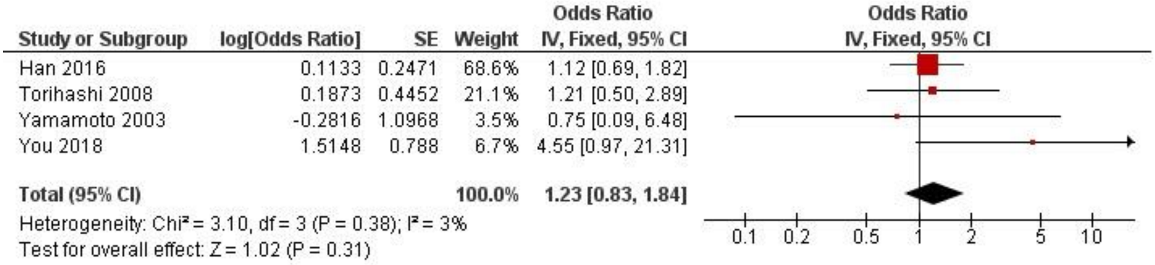

Forest plot of comparison: 1 Risk factor of Chronic Subdural Hematoma recurrence: Clinical History, Risk factor: 1.10 Heart disease.

Figure S9.1 Forest plot comparison for risk factor of chronic subdural hematoma recurrence, Risk factor: 1.3 Hypertension.

Study or Subgroup	log[Odds Ratio]	SE	Weight	Odds Ratio IV, Fixed, 95% CI
Andersen 2019	0.3365	0.1717	24.5%	1.40 [1.00, 1.96]
Chon 2012	-0.0632	0.2541	11.2%	0.94 [0.57, 1.54]
dos Santos 2019	-0.1394	0.3572	5.7%	0.87 [0.43, 1.75]
Han 2016	-0.1508	0.201	17.9%	0.86 [0.58, 1.28]
Kim 2015	0.4507	0.3773	5.1%	1.57 [0.75, 3.29]
Motoie 2018	0.3293	0.2161	15.5%	1.39 [0.91, 2.12]
Shen2019	0.1039	0.2619	10.5%	1.11 [0.66, 1.85]
Torihashi 2008	-0.2863	0.3766	5.1%	0.75 [0.36, 1.57]
Yamamoto 2003	2.4314	0.8959	0.9%	11.37 [1.96, 65.85]
You 2018	-0.5371	0.4521	3.5%	0.58 [0.24, 1.42]
**Total (95% CI)**			**100.0%**	**1.12 [0.95, 1.32]**

Heterogeneity: Chi² = 16.09, df = 9 (P = 0.07); I² = 44%
Test for overall effect: Z = 1.34 (P = 0.18)

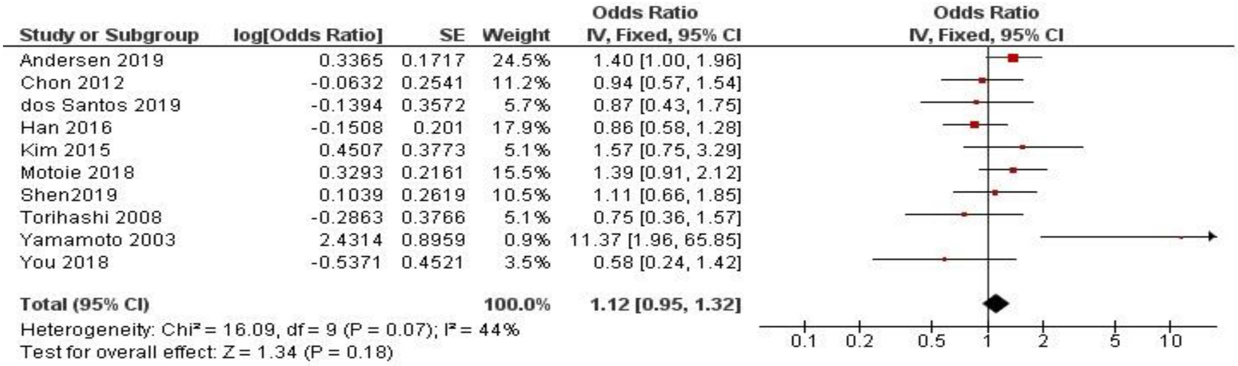

Forest plot of comparison: 1 Risk factor of Chronic Subdural Hematoma recurrence, Risk factor: 1.3 Hypertension.

Figure S20. Forest plot comparison for risk factor of chronic subdural hematoma recurrence, Risk factor: 1.9 Liver disease.

Study or Subgroup	log[Odds Ratio]	SE	Weight	Odds Ratio IV, Fixed, 95% CI
Chon 2012	0.8873	0.3335	37.1%	2.43 [1.26, 4.67]
dos Santos 2019	0.8931	0.7803	6.8%	2.44 [0.53, 11.27]
Han 2016	0.3853	0.2795	52.9%	1.47 [0.85, 2.54]
Yamamoto 2003	0.383	1.1305	3.2%	1.47 [0.16, 13.45]
**Total (95% CI)**			**100.0%**	**1.83 [1.23, 2.73]**

Heterogeneity: Chi² = 1.51, df = 3 (P = 0.68); I² = 0%
Test for overall effect: Z = 2.98 (P = 0.003)

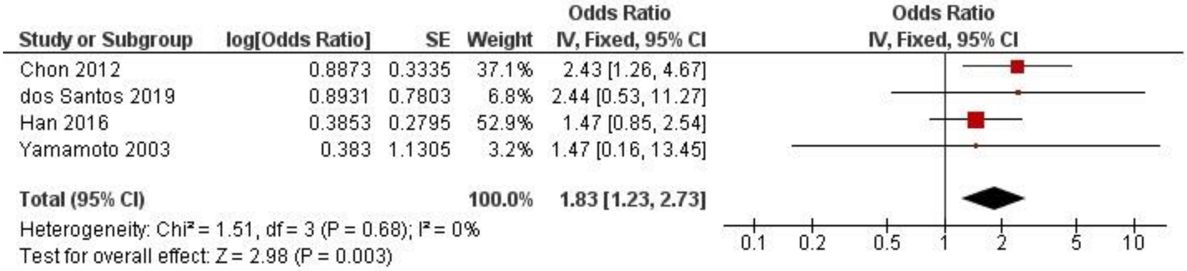

Forest plot of comparison: 1 Risk factor of Chronic Subdural Hematoma recurrence, Risk factor: 1.9 Liver disease.

Figure S11. Forest plot comparison for risk factor of chronic subdural hematoma recurrence, Risk factor: 1.11 Malignancy.

Study or Subgroup	log[Odds Ratio]	SE	Weight	Odds Ratio IV, Fixed, 95% CI
Han 2016	-0.1393	0.3716	37.6%	0.87 [0.42, 1.80]
Kim 2015	0.9652	0.534	18.2%	2.63 [0.92, 7.48]
Motoie 2018	-0.0619	0.3429	44.2%	0.94 [0.48, 1.84]
**Total (95% CI)**			**100.0%**	**1.10 [0.70, 1.72]**

Heterogeneity: Chi² = 3.26, df = 2 (P = 0.20); I² = 39%
Test for overall effect: Z = 0.42 (P = 0.67)

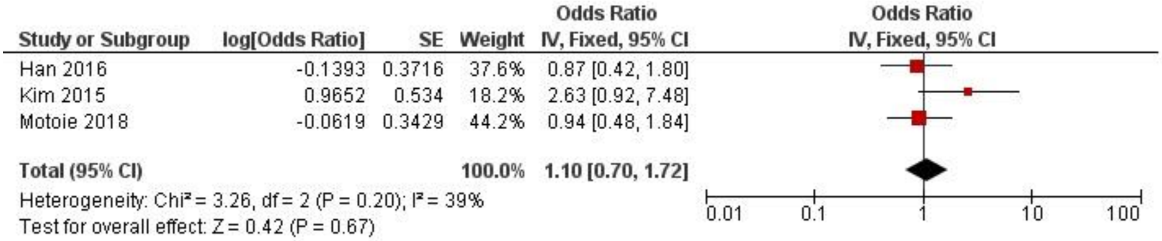

Forest plot of comparison: 1 Risk factor of Chronic Subdural Hematoma recurrence, Risk factor: 1.11 Malignancy.

Figure S12. Forest plot comparison for risk factor of chronic subdural hematoma recurrence: Clinical history, Risk factor: 1.6 Obesity

Study or Subgroup	log[Odds Ratio]	SE	Weight	Odds Ratio IV, Fixed, 95% CI
Han 2016	0.5933	0.2588	100.0%	1.81 [1.09, 3.01]
**Total (95% CI)**			**100.0%**	**1.81 [1.09, 3.01]**

Heterogeneity: Not applicable
Test for overall effect: Z = 2.29 (P = 0.02)

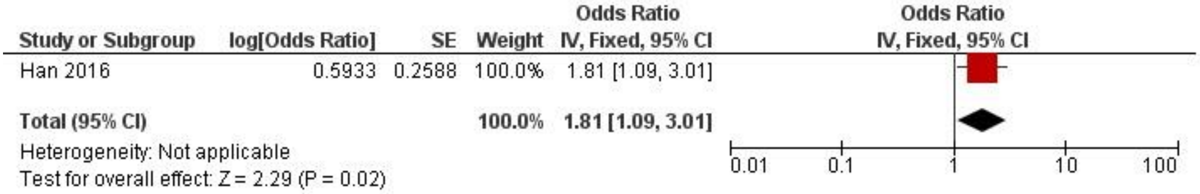

Forest plot of comparison: 1 Risk factor of Chronic Subdural Hematoma recurrence: Clinical History, Risk factor: 1.6 Obesity.

Figure S13. Forest plot comparison for risk factor of chronic subdural hematoma recurrence, Risk factor: 1.14 Seizure.

Study or Subgroup	log[Odds Ratio]	SE	Weight	Odds Ratio IV, Fixed, 95% CI
Chon 2012	0.9416	0.3831	57.7%	2.56 [1.21, 5.43]
Hammer 2017	1.118	1.0383	7.9%	3.06 [0.40, 23.41]
Kim 2015	0.9502	1.1348	6.6%	2.59 [0.28, 23.91]
Leroy 2015	0.4149	0.7003	17.3%	1.51 [0.38, 5.97]
Yamamoto 2003	2.4314	0.8959	10.6%	11.37 [1.96, 65.85]
**Total (95% CI)**			**100.0%**	**2.78 [1.57, 4.92]**

Heterogeneity: Chi² = 3.28, df = 4 (P = 0.51); I² = 0%
Test for overall effect: Z = 3.51 (P = 0.0004)

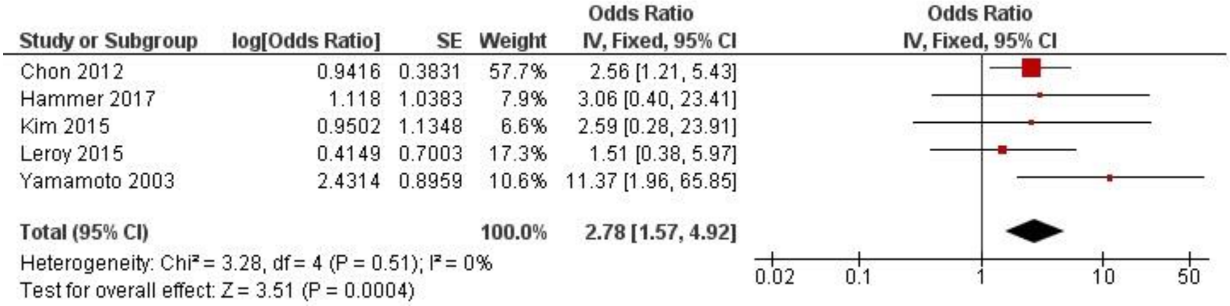

Forest plot of comparison: 1 Risk factor of Chronic Subdural Hematoma recurrence, Risk factor: 1.14 Seizure.

Figure S14. Forest plot comparison for risk factor of chronic subdural hematoma recurrence, Risk factor: 1.12 Smoking.

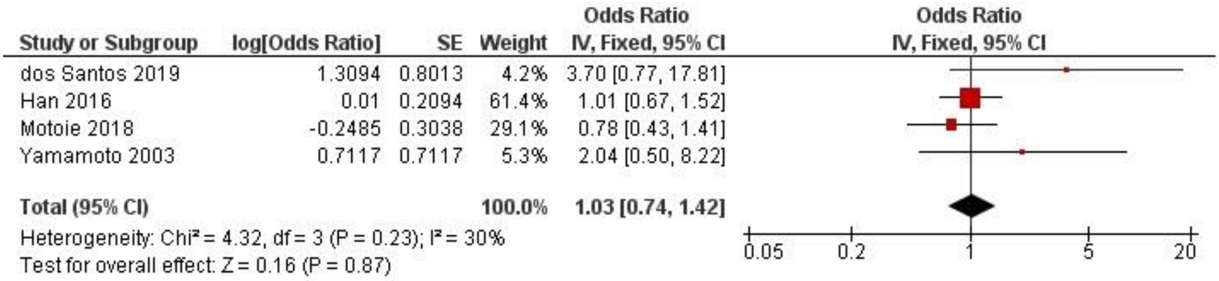

Forest plot of comparison: 1 Risk factor of Chronic Subdural Hematoma recurrence, Risk factor: 1.12 Smoking.

Figure S15. Forest plot comparison for risk factor of chronic subdural hematoma recurrence, Risk factor: 1.13 Stroke.

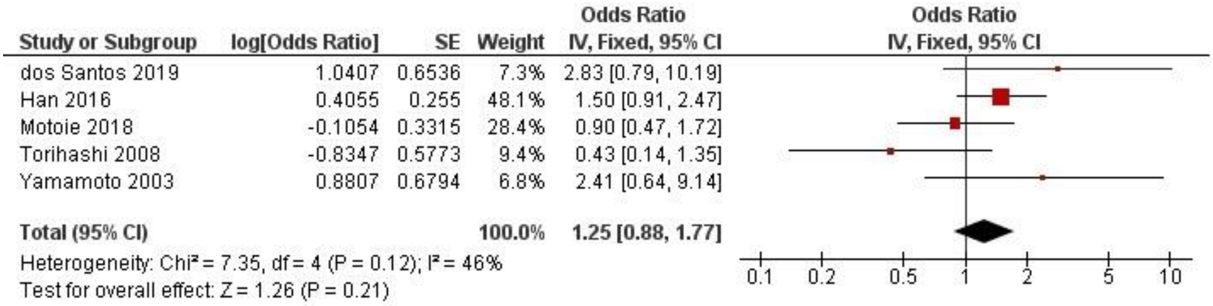

Forest plot of comparison: 1 Risk factor of Chronic Subdural Hematoma recurrence, Risk factor: 1.13 Stroke

Figure S16. Forest plot comparison for risk factor of chronic subdural hematoma recurrence on CT scan, Risk factor: 2.10 bilateral hematoma

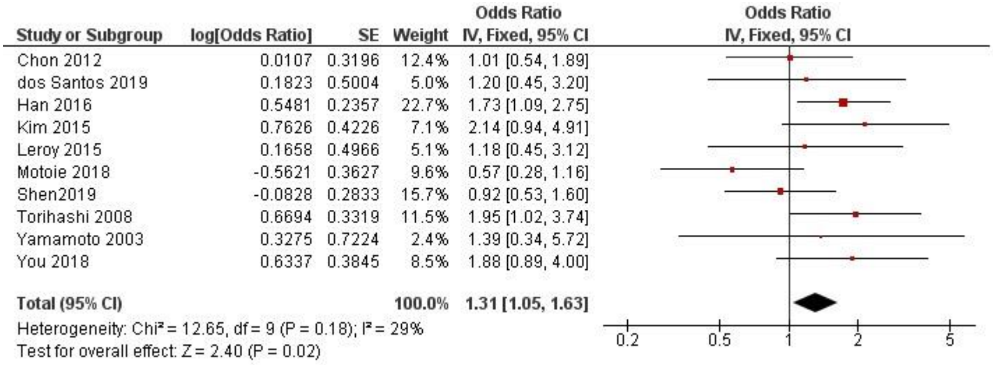

Forest plot of comparison: 2 Risk factor of Chronic Subdural Hematoma recurrence on CT scan, Risk factor: 2.10 Bilateral hematoma.

Figure S17. Forest plot comparison for risk factor of chronic subdural hematoma recurrence on CT scan, Risk factor: 2.11 severe brain atrophy.

Study or Subgroup	log[Odds Ratio]	SE	Weight	Odds Ratio IV, Fixed, 95% CI
Andersen 2019	0.6931	0.2198	59.4%	2.00 [1.30, 3.08]
Chon 2012	0.6974	0.3428	24.4%	2.01 [1.03, 3.93]
Oishi 2001	1.8563	0.726	5.4%	6.40 [1.54, 26.56]
Shen2019	2.5741	0.5152	10.8%	13.12 [4.78, 36.01]
**Total (95% CI)**			**100.0%**	**2.61 [1.88, 3.64]**

Heterogeneity: Chi² = 13.40, df = 3 (P = 0.004); I² = 78%
Test for overall effect: Z = 5.67 (P < 0.00001)

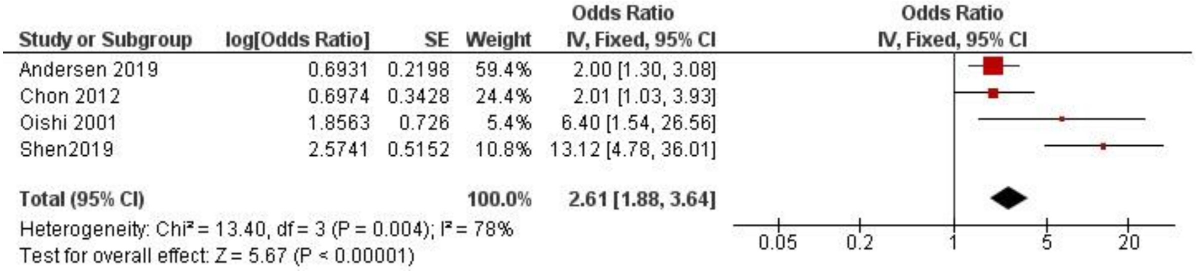

Forest plot of comparison: 2 Risk factor of Chronic Subdural Hematoma recurrence on CT scan, Risk factor: 2.11 Severe brain atrophy.

Figure S18. Forest plot comparison for risk factor of chronic subdural hematoma recurrence on CT scan, Risk factor: 2.3 Internal architecture of the hematoma: homogeneous.

Study or Subgroup	log[Odds Ratio]	SE	Weight	Odds Ratio IV, Fixed, 95% CI
Andersen 2019	0.5878	0.2999	22.6%	1.80 [1.00, 3.24]
Chon 2012	0.1735	0.34	17.6%	1.19 [0.61, 2.32]
Hammer 2017	-0.9105	0.525	7.4%	0.40 [0.14, 1.13]
Leroy 2015	0.0933	0.5497	6.7%	1.10 [0.37, 3.22]
Ohba 2012	0.822	0.8933	2.5%	2.28 [0.40, 13.10]
Shen2019	0.5898	0.2632	29.3%	1.80 [1.08, 3.02]
You 2018	0.3962	0.3813	14.0%	1.49 [0.70, 3.14]
**Total (95% CI)**			**100.0%**	**1.42 [1.07, 1.88]**

Heterogeneity: Chi² = 8.00, df = 6 (P = 0.24); I² = 25%
Test for overall effect: Z = 2.47 (P = 0.01)

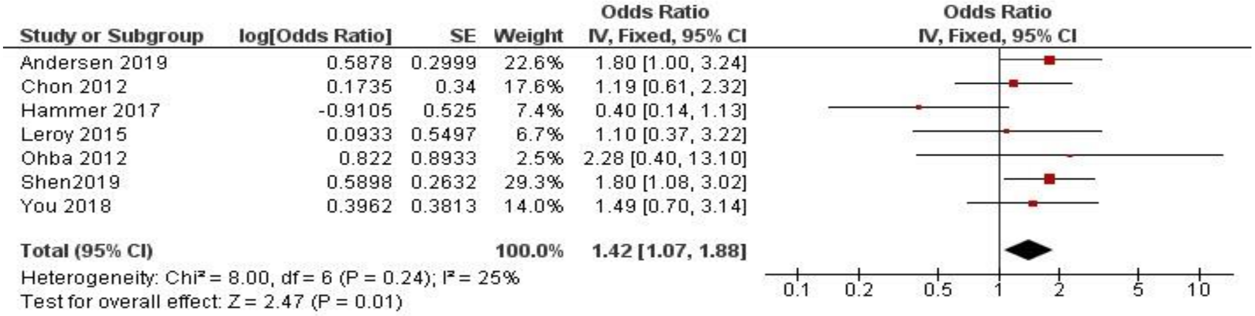

Forest plot of comparison: 2 Risk factor of Chronic Subdural Hematoma recurrence on CT scan, Risk factor: 2.3 Internal architecture of the hematoma: Homogeneous.

Figure S19. Forest plot comparison for risk factor of chronic subdural hematoma recurrence on CT scan, Risk factor: 2.9 hematoma density: hyperdensity.

Study or Subgroup	log[Odds Ratio]	SE	Weight	Odds Ratio IV, Fixed, 95% CI
Chon 2012	-1.722	1.0329	5.9%	0.18 [0.02, 1.35]
Hammer 2017	0.3655	0.9106	7.5%	1.44 [0.24, 8.59]
Oishi 2001	0.3042	0.6623	14.3%	1.36 [0.37, 4.96]
Shen2019	-0.0028	0.3569	49.1%	1.00 [0.50, 2.01]
You 2018	1.3581	0.5184	23.3%	3.89 [1.41, 10.74]
**Total (95% CI)**			**100.0%**	**1.33 [0.81, 2.17]**

Heterogeneity: Chi² = 8.72, df = 4 (P = 0.07); I² = 54%
Test for overall effect: Z = 1.14 (P = 0.26)

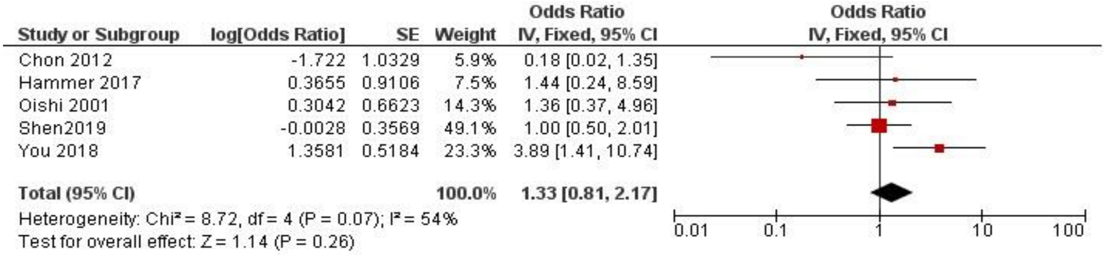

Forest plot of comparison: 2 Risk factor of Chronic Subdural Hematoma recurrence on CT scan, Risk factor: 2.9 Hematoma density: Hyperdensity.

Figure S20. Forest plot comparison for risk factor of chronic subdural hematoma recurrence on CT scan, Risk factor: 2.7 hematoma density: hyperdensity.

Study or Subgroup	log[Odds Ratio]	SE	Weight	Odds Ratio IV, Fixed, 95% CI
Chon 2012	-0.7167	0.4541	24.0%	0.49 [0.20, 1.19]
Hammer 2017	-1.4663	0.5718	15.2%	0.23 [0.08, 0.71]
Kim 2015	0.397	0.4049	30.2%	1.49 [0.67, 3.29]
Oishi 2001	-1.269	1.4733	2.3%	0.28 [0.02, 5.05]
You 2018	0.557	0.4185	28.3%	1.75 [0.77, 3.96]
**Total (95% CI)**			**100.0%**	**0.86 [0.56, 1.34]**

Heterogeneity: Chi² = 12.11, df = 4 (P = 0.02); I² = 67%
Test for overall effect: Z = 0.66 (P = 0.51)

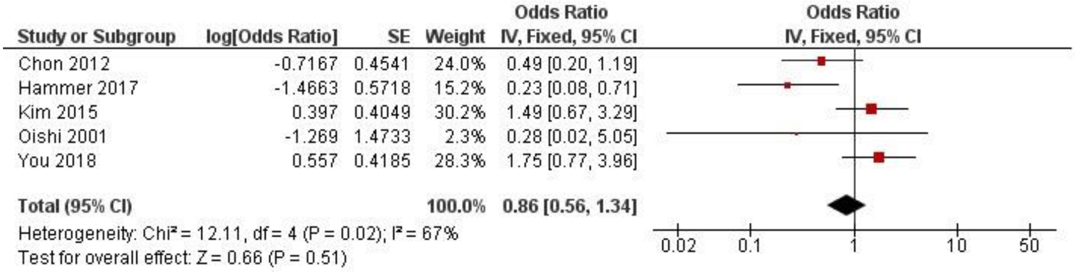

Forest plot of comparison: 2 Risk factor of Chronic Subdural Hematoma recurrence on CT scan, Risk factor : 2.7 Hematoma density: Hypodensity.

Figure S21. Forest plot comparison for risk factor of chronic subdural hematoma recurrence on CT scan, Risk factor: 2.8 hematoma density: Isodensity.

Study or Subgroup	log[Odds Ratio]	SE	Weight	Odds Ratio IV, Fixed, 95% CI
Chon 2012	-0.2302	0.2419	43.9%	0.79 [0.49, 1.28]
Hammer 2017	-1.4663	1.5011	1.1%	0.23 [0.01, 4.37]
Kim 2015	0.7943	0.391	16.8%	2.21 [1.03, 4.76]
Oishi 2001	-1.7047	1.0713	2.2%	0.18 [0.02, 1.48]
Shen2019	-0.5123	0.3049	27.6%	0.60 [0.33, 1.09]
You 2018	-0.998	0.5568	8.3%	0.37 [0.12, 1.10]
**Total (95% CI)**			**100.0%**	**0.78 [0.57, 1.07]**

Heterogeneity: Chi² = 12.18, df = 5 (P = 0.03); I² = 59%
Test for overall effect: Z = 1.54 (P = 0.12)

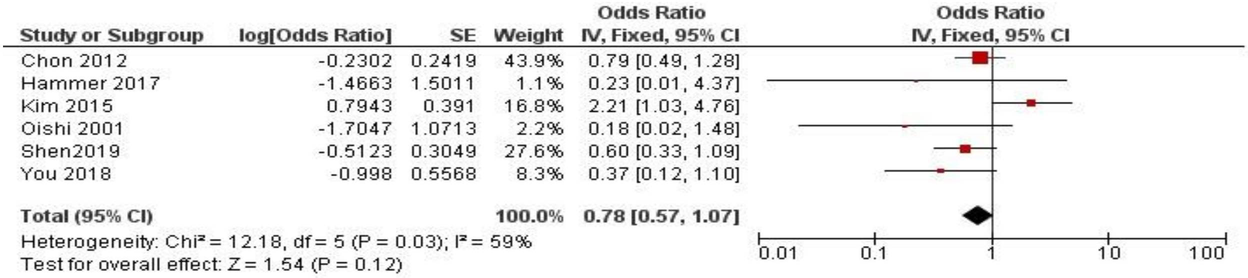

Forest plot of comparison: 2 Risk factor of Chronic Subdural Hematoma recurrence on CT scan, Risk factor : 2.8 Hematoma density: Isodensity.

Figure S22. Forest plot comparison for risk factor of chronic subdural hematoma recurrence on CT scan, Risk factor: 2.4 Internal architecture of the hematoma: Laminar.

Study or Subgroup	log[Odds Ratio]	SE	Weight	Odds Ratio IV, Fixed, 95% CI
Chon 2012	0.677	0.2509	73.2%	1.97 [1.20, 3.22]
Hammer 2017	0.1423	0.8827	5.9%	1.15 [0.20, 6.50]
Ohba 2012	1.2261	0.9906	4.7%	3.41 [0.49, 23.75]
Shen2019	-0.8109	0.6157	12.2%	0.44 [0.13, 1.49]
You 2018	-0.361	1.0774	4.0%	0.70 [0.08, 5.76]
**Total (95% CI)**			**100.0%**	**1.57 [1.03, 2.39]**

Heterogeneity: Chi² = 6.31, df = 4 (P = 0.18); I² = 37%
Test for overall effect: Z = 2.09 (P = 0.04)

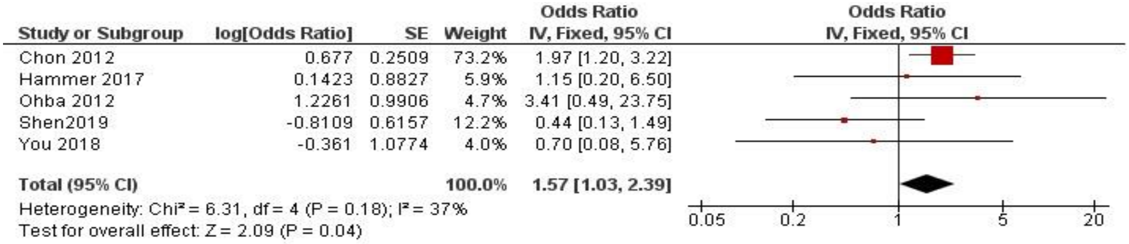

Forest plot of comparison: 2 Risk factor of Chronic Subdural Hematoma recurrence on CT scan, Risk factor: 2.4 Internal architecture of the hematoma: Laminar

Figure S23. Forest plot comparison for risk factor of chronic subdural hematoma recurrence on CT scan, Risk factor: 2.1 Midline shift>10mm (1cm).

Study or Subgroup	log[Odds Ratio]	SE	Weight	Odds Ratio IV, Fixed, 95% CI
Andersen 2019	0.1823	0.1468	32.9%	1.20 [0.90, 1.60]
Chon 2012	1.9003	0.2438	11.9%	6.69 [4.15, 10.78]
Han 2016	0.207	0.2007	17.6%	1.23 [0.83, 1.82]
Kim 2015	1.3211	0.3867	4.7%	3.75 [1.76, 8.00]
Leroy 2015	1.0941	0.516	2.7%	2.99 [1.09, 8.21]
Ohba 2012	0.2539	0.189	19.8%	1.29 [0.89, 1.87]
Shen2019	1.0852	0.4044	4.3%	2.96 [1.34, 6.54]
Yamamoto 2003	3.247	0.8337	1.0%	25.71 [5.02, 131.77]
You 2018	0.3269	0.373	5.1%	1.39 [0.67, 2.88]
**Total (95% CI)**			**100.0%**	**1.75 [1.49, 2.07]**

Heterogeneity: Chi² = 59.97, df = 8 (P < 0.00001); I² = 87%
Test for overall effect: Z = 6.67 (P < 0.00001)

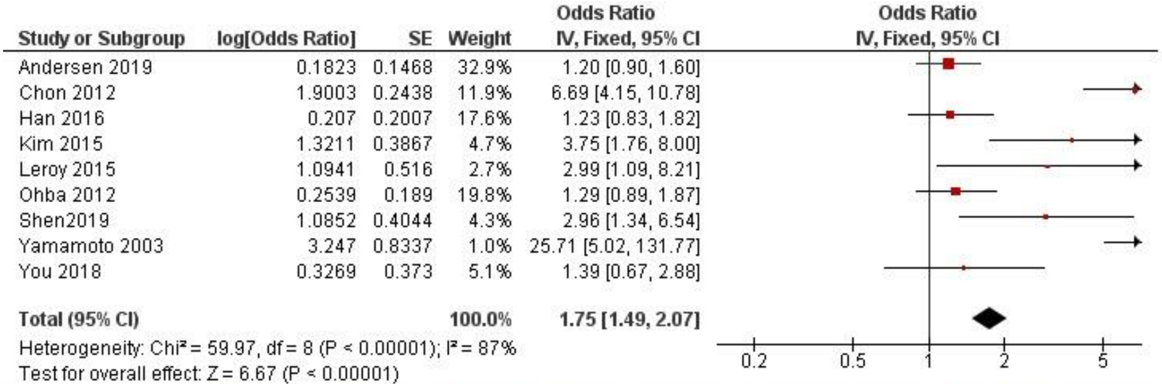

Forest plot of comparison: 2 Risk factor of Chronic Subdural Hematoma recurrence on CT scan, Risk factor: 2.1 Midline Shift >10 mm (1cm).

Figure S24. Forest plot comparison for risk factor of chronic subdural hematoma recurrence on CT scan, Outcome: 2.12 Postoperative pneumocephalus.

Study or Subgroup	log[Odds Ratio]	SE	Weight	Odds Ratio IV, Fixed, 95% CI
Ohba 2012	0.5309	0.7018	14.2%	1.70 [0.43, 6.73]
Oishi 2001	1.4618	0.8141	10.5%	4.31 [0.87, 21.27]
Shen2019	0.8416	0.3238	66.6%	2.32 [1.23, 4.38]
Yamamoto 2003	0.793	0.8983	8.7%	2.21 [0.38, 12.85]
**Total (95% CI)**			**100.0%**	**2.36 [1.41, 3.96]**

Heterogeneity: Chi² = 0.78, df = 3 (P = 0.86); I² = 0%
Test for overall effect: Z = 3.25 (P = 0.001)

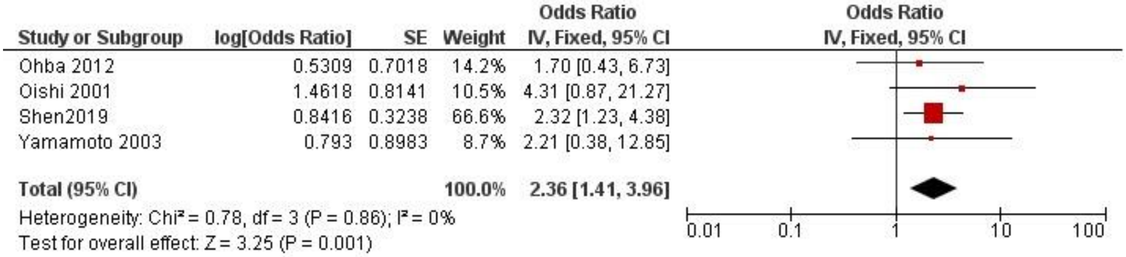

Forest plot of comparison: 2 Risk factor of Chronic Subdural Hematoma recurrence on CT scan, outcome: 2.12 Postoperative pneumocephalus.

Figure S25. Forest plot comparison for risk factor of chronic subdural hematoma recurrence on CT scan, Risk factor: 2.5 Internal architecture of the hematoma: Separated.

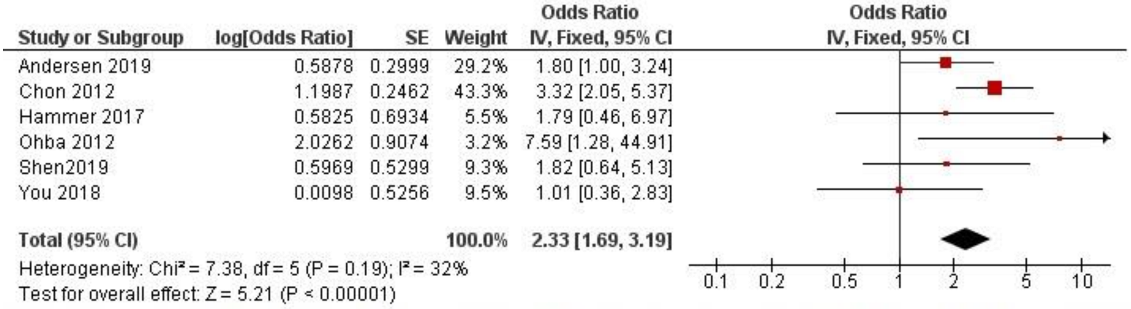

Study or Subgroup	log[Odds Ratio]	SE	Weight	Odds Ratio IV, Fixed, 95% CI
Andersen 2019	0.5878	0.2999	29.2%	1.80 [1.00, 3.24]
Chon 2012	1.1987	0.2462	43.3%	3.32 [2.05, 5.37]
Hammer 2017	0.5825	0.6934	5.5%	1.79 [0.46, 6.97]
Ohba 2012	2.0262	0.9074	3.2%	7.59 [1.28, 44.91]
Shen2019	0.5969	0.5299	9.3%	1.82 [0.64, 5.13]
You 2018	0.0098	0.5256	9.5%	1.01 [0.36, 2.83]
**Total (95% CI)**			**100.0%**	**2.33 [1.69, 3.19]**

Heterogeneity: Chi² = 7.38, df = 5 (P = 0.19); I² = 32%
Test for overall effect: Z = 5.21 (P < 0.00001)

Forest plot of comparison: 2 Risk factor of Chronic Subdural Hematoma recurrence on CT scan, Risk factor: 2.5 Internal architecture of the hematoma:Separated.

Figure S26. Forest plot comparison for risk factor of chronic subdural hematoma recurrence on CT scan, Risk factor: 2.6 Internal architecture of the hematoma: Trabecular.

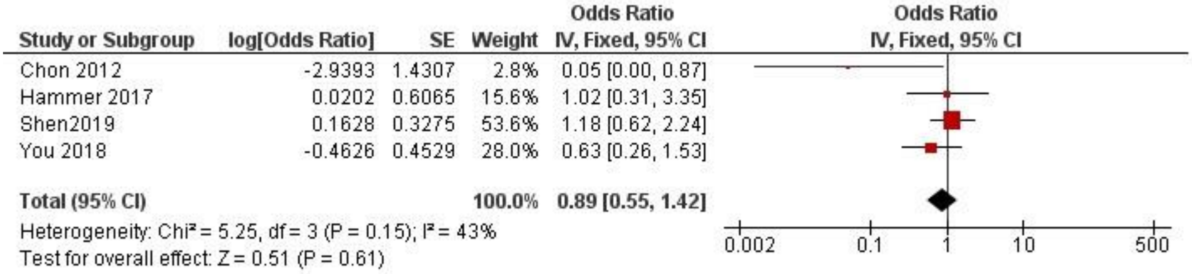

Study or Subgroup	log[Odds Ratio]	SE	Weight	Odds Ratio IV, Fixed, 95% CI
Chon 2012	-2.9393	1.4307	2.8%	0.05 [0.00, 0.87]
Hammer 2017	0.0202	0.6065	15.6%	1.02 [0.31, 3.35]
Shen2019	0.1628	0.3275	53.6%	1.18 [0.62, 2.24]
You 2018	-0.4626	0.4529	28.0%	0.63 [0.26, 1.53]
**Total (95% CI)**			**100.0%**	**0.89 [0.55, 1.42]**

Heterogeneity: Chi² = 5.25, df = 3 (P = 0.15); I² = 43%
Test for overall effect: Z = 0.51 (P = 0.61)

Forest plot of comparison: 2 Risk factor of Chronic Subdural Hematoma recurrence on CT scan, Risk factor: 2.6 Internal architecture of the hematoma:Trabecular.

Figure S27. Forest plot comparison for risk factor of chronic subdural hematoma recurrence on CT scan, Risk factor: 2.2 width of hematoma (>20).

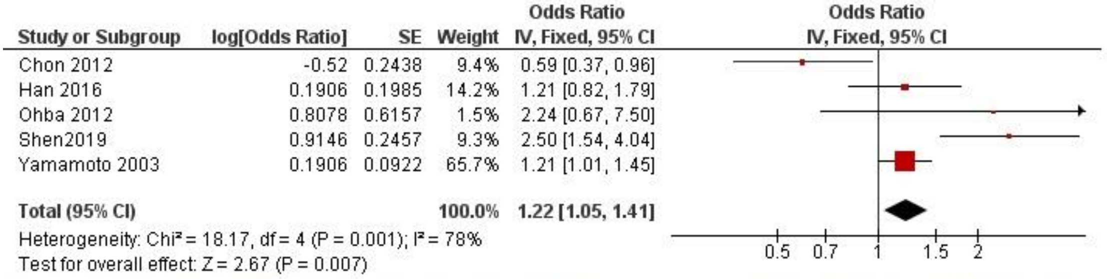

Study or Subgroup	log[Odds Ratio]	SE	Weight	Odds Ratio IV, Fixed, 95% CI
Chon 2012	-0.52	0.2438	9.4%	0.59 [0.37, 0.96]
Han 2016	0.1906	0.1985	14.2%	1.21 [0.82, 1.79]
Ohba 2012	0.8078	0.6157	1.5%	2.24 [0.67, 7.50]
Shen2019	0.9146	0.2457	9.3%	2.50 [1.54, 4.04]
Yamamoto 2003	0.1906	0.0922	65.7%	1.21 [1.01, 1.45]
**Total (95% CI)**			**100.0%**	**1.22 [1.05, 1.41]**

Heterogeneity: Chi² = 18.17, df = 4 (P = 0.001); I² = 78%
Test for overall effect: Z = 2.67 (P = 0.007)

Forest plot of comparison: 2 Risk factor of Chronic Subdural Hematoma recurrence on CT scan, Risk factor: 2.2 Width of hematoma (>20mm).
